# Plasmacytoid Dendritic Cells in Patients with MGUS and Multiple Myeloma

**DOI:** 10.3390/jcm10163717

**Published:** 2021-08-20

**Authors:** Andrea Knight, Lucie Rihova, Romana Kralova, Miroslav Penka, Zdenek Adam, Ludek Pour, Martin Piskacek, Roman Hajek

**Affiliations:** 1 Department of Pathological Physiology, Faculty of Medicine, Masaryk University, 625 00 Brno, Czech Republic; piskacek@med.muni.cz; 2 Department of Hematology, University Hospital Brno, 625 00 Brno, Czech Republic; Rihova.Lucie@fnbrno.cz (L.R.); kralovar@email.cz (R.K.); penka.miroslav@fnbrno.cz (M.P.); 3 Department of Internal Medicine, Hematology and Oncology, University Hospital Brno, 625 00 Brno, Czech Republic; adam.zdenek@fnbrno.cz (Z.A.); pour.ludek@fnbrno.cz (L.P.); 4 Department of Hemato-Oncology, University Hospital Ostrava, 708 00 Ostrava, Czech Republic; roman.hajek@fno.cz; 5 Faculty of Medicine, University of Ostrava, 701 03 Ostrava, Czech Republic

**Keywords:** plasmacytoid dendritic cells, MGUS, multiple myeloma, immunosuppressive tumor microenvironment

## Abstract

Background: Plasmacytoid dendritic cells (pDCs) play prominent roles in mediating innate and adaptive immune responses. However, it is unclear how pDCs contribute to the immunosuppressive tumor microenvironment described in multiple myeloma (MM). Methods: Newly diagnosed myeloma patients (MM, *n* = 37) were analyzed to determine the pDC counts in comparison to peripheral blood (PB, *n* = 53) and bone marrow (BM, *n* = 10) samples of age-matched healthy donors (HD) using flow cytometry. Second, proliferation of myeloma tumor cells in the presence of freshly isolated pDCs was examined. Third, production of IFNα by pDCs co-cultured with MM cells was determined by intracellular staining. Results: We found a highly significant reduction of circulating pDCs (*p* < 0.0001) and in bone marrow (*p* < 0.0001) of MM patients compared to HD. We also observed a significant decrease of pDCs (*p* = 0.004) in BM in patients with monoclonal gammopathy of undetermined significance (MGUS, *n* = 12). Importantly, we determined that pDCs promote proliferation specifically of MM cells and not the stromal cells and that pDCs secrete IFNα upon co-culture with MM tumor cells. Conclusions: Our results show altered pDC frequencies in the BM microenvironment in MGUS and MM patients at diagnosis. We showed the tumor-promoting function of pDCs that may mediate immune deficiencies affecting long-term disease control and treatment outcome.

## 1. Introduction

Plasmacytoid dendritic cells (pDCs) are a distinct lineage of bone marrow-derived cells that reside mainly in blood and lymphoid organs but can be also found in sites of infection and inflammation. Human pDCs have a unique cell surface phenotype; they lack B, T, myeloid, and NK lineage markers and express CD123 (IL-3Rα), CD303 (BDCA-2), CD304 (BDCA-4), CD4, CD45RA, ILT3 and ILT7 [1,2]. Moreover, they express pathogen recognition receptors TLR-7 and TLR-9 mainly residing in the endosomes responding to viral RNA and DNA upon cell infection. This leads to secretion of large amounts of cytokines, particularly type I interferons, including INFα, resulting in regulation of inflammation and activation of NK cell cytotoxic activity, linking innate with adaptive immunity [3,4,5,6]. PDCs are capable of activating CD4 helper and regulatory T cells and CD8 cytotoxic T cells, they stimulate B-cell activation, induce B cells to differentiate into plasma cells and produce antibodies [7,8,9]. The role of pDCs in tumor immunity is not yet fully known. Several reports have shown that tumor-infiltrating pDCs in solid tumors were predominantly pro-tumorigenic with reduced IFNα secretion contributing to the immunosuppressive tumor microenvironment [10,11]. Importantly, pDCs were identified as negative prognostic markers and positive predictors of disease progression in breast [12,13], ovarian [14,15], melanoma [16] and gastric [17] cancers. However, there are only sporadic reports concerning the role of pDCs in hematological malignancies.

Multiple myeloma (MM) is characterized by the clonal expansion and accumulation of malignant plasma cells in the bone marrow, producing high amounts of monoclonal immunoglobulin [18,19]. It is considered the second most common blood cancer and remains incurable due to the development of cancer cell intrinsic mechanisms [20,21]. It is accepted that MM cells induce direct and also indirect signaling sequelae in the BM, supporting tumor cell proliferation, survival and drug resistance. The mechanisms of immune escape together with a decrease in effective immune cell infiltration and the accumulation of immunosuppressive cells including tumor-associated macrophages (TAMs), myeloid-derived suppressor cells (MDSCs), T regulatory cells (Tregs), and tumor-associated neutrophils (TANs) influence the anti-tumor immune responses [22]. The innate and adaptive immune cells in the BM microenvironment harbor both tumor-promoting and tumor-suppressing activities, which may predict patient outcome [23,24].

An early report showed reduced pDC numbers in MM patients compared to healthy controls in addition to impaired function of pDCs in regard to antigen presentation and cytokine production [25]. Others found increased numbers of pDCs in bone marrow from MM patients and a pathophysiologic role of pDC where it supports MM tumor cell survival and growth [26]. Importantly, the authors showed that pDCs were relatively resistant to bortezomib compared to MM cells, similar to lenalidomide, which did not decrease the viability of pDCs. Moreover, pDCs triggered increased DNA synthesis in MM cells even in the presence of lenalidomide [26].

In this study, we first aimed to determine the pDC numbers in newly diagnosed MM patients, determine the proliferation of myeloma tumor cells in the presence of freshly isolated pDCs, and examine the pDC function including the IFNα production in the presence of MM cells. Second, we analyzed bone marrow from patients with monoclonal gammopathy of undetermined significance (MGUS), which is characterized as a pre-existing condition to the majority of MM patients. On average, about 1% of MGUS patients will go on to develop MM each year [27,28].

Our results show significant reduction of pDCs in peripheral blood and bone marrow of MM patients compared to HD. In addition, we observed a significant decrease of pDCs in BM in patients with MGUS. Furthermore, we found that pDCs promote MM cell proliferation and secrete IFNα upon co-culture with MM tumor cells. These findings confirm aberrant roles of pDCs within the BM tumor microenvironment in myeloma.

## 2. Materials and Methods

### 2.1. Patients

This study enrolled consecutive patients attending the clinic and two cohorts of newly diagnosed MM patients (*n* = 37) and MGUS patients (*n* = 12) without any prior treatment. No selection criteria were used. No smoldering myeloma patients were included. Patient’s clinical characteristics are listed in Table 1. Peripheral blood (PB) and paired bone marrow (BM) samples were obtained from patients at the Department of Internal Medicine, Hematology and Oncology, Faculty Hospital Brno, in accordance with the Declaration of Helsinki and approved protocols by the Institutional review board and ethics committee of Masaryk University, Brno.

### 2.2. Healthy Donors

Fresh peripheral blood (PB) and bone marrow (BM) samples from healthy donors (*n* = 10) undergoing post-degenerative knee prosthesis implantation at the local Traumatology and Orthopedic Centre Brno were obtained. Additionally, buffy coats from 43 age-matched healthy donors (HD) were collected from the Transfusion and Tissue Bank, Faculty Hospital Brno after obtaining informed consent. The cohort of healthy donors included 19 females and 34 males, with a median age of 61 years (range, 52–85 years). All participants gave written informed consent. Samples were obtained and processed within 2 h of collection. Peripheral blood mononuclear cells (PBMC) and bone marrow mononuclear cells (BMMC) were isolated by standard density gradient centrifugation using Lymphoprep (Stem Cell Technologies, Cambridge, UK) following the manufacturer’s instructions.

### 2.3. Flow Cytometry Phenotyping

Single-cell suspension of PBMC and BMMC cells were stained with monoclonal antibodies purchased from Miltenyi Biotec (Germany) including CD123 (clone AC145), CD303 (BDCA-2, clone AC144), CD304 (Neurophilin-1/BDCA-4, clone AD5-17F6), HLA-DR (clone Immu-357, Beckman Coulter), CD45 (clone J33, Beckman Coulter), CD45RA (clone HI100, BD Pharmingen^TM^), CD33 (clone P67.6, BD Biosciences) and CD38 (clone HIT2, Exbio, Prague, Czech Republic). Samples were acquired using FACS Canto^®^ (BD Biosciences) and data were analyzed using FlowJo^®^ software (FlowJo, Ashland, OR, USA). Forward and side scatter gating were used to discriminate live cells from dead cells and were derived from SSC vs. FSC gated bulk PBMCs or BMMCs with doublet exclusion (FSC-A vs. FCS-H). To determine the placement of the gates, appropriate fluorescence minus one (FMO) and unstained controls were used.

### 2.4. Cell Isolation of Primary pDCs

Plasmacytoid dendritic cells were freshly isolated from PBMCs by a direct magnetic labeling using the Diamond Plasmacytoid Dendritic Cell Isolation Kit II according to the manufacturer’s instructions (Miltenyi Biotec). Purity of sorted pDCs was confirmed by flow cytometry by staining with CD123 and CD303 antibodies and was routinely over 85%.

### 2.5. Cell Culture

Multiple myeloma cell line U266 was purchased from DSMZ (German Collection of Microorganisms and Cell Culture GmbH, Germany). KMS-11, OPM-2 and LP-1 (kind gift from Dr Krejci, Department of Biology, Masaryk University Brno) were cultured in RPMI-1640 containing 10% fetal bovine serum (FBS) and 2% penicillin/streptomycin (all Thermo Fisher Scientific). Human bone marrow stromal cell line HS-5 was purchased from ATCC (American Type Culture Collection, Manassas, VA, USA) and maintained in Dulbecco’s Modified Eagle’s Medium (DMEM) supplemented with 10% FBS, 2 mM L-glutamine and 2% penicillin/streptomycin (Thermo Fisher Scientific). All cells were grown at 37 °C in 5% CO_2_ atmosphere up to 70–80% confluence. The adherent cell line HS-5 was harvested by using gentle dissociation solution TrypLE (Gibco, Thermo Fisher Scientific) and counted by using Trypan blue exclusion.

### 2.6. Intracellular Cytokine Detection

Production of IFNα by pDCs was determined by intracellular staining using anti-IFNα monoclonal antibody (clone 7N4-1, BD Pharmingen^TM^) and the Intracellular Fixation & Permeabilization Buffer Kit (Thermo Fisher Scientific). Freshly sorted pDCs (100,000) were stimulated in 24 well plates (Corning^®^ Costar^®^ cell culture plate) with MM tumor cells and HS-5 cells at a 1:5 ratio. Brefeldin A (Sigma) was used at 10 μg/mL per 10^6^ cells. Stimulation of pDCs alone with PMA (Phorbol 12-myristate 13-acetate, Sigma) and CI (Calcium Ionophore, Sigma) at 0.25 μg/mL and 0.05 μg/mL per 10^5^ cells, respectively, were determined as positive controls.

### 2.7. Statistical Analyses

Statistical analyses were performed using Prism^®^ (GraphPad Software, La Jolla, CA, USA). Comparisons between two independent groups were done using the non-parametric Mann–Whitney test and the unpaired *t* test. Statistical significance was determined by a *p* value of less than 0.05.

## 3. Results

### 3.1. Plasmacytoid Dendritic Cells in Myeloma Patients Compared to Healthy Donors

**Figure 1:**
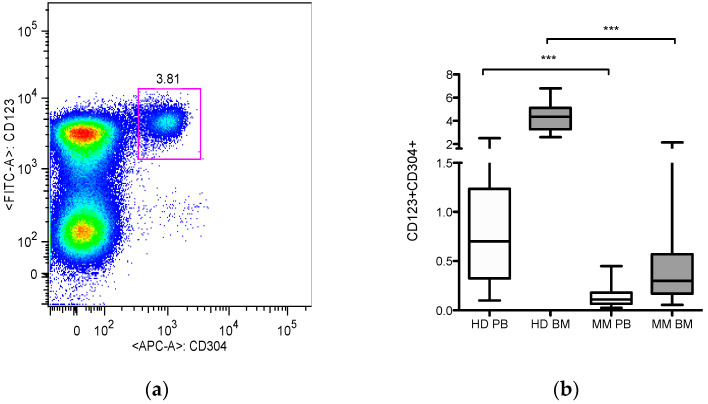
Plasmacytoid dendritic cells in healthy donors and multiple myeloma patients: (**a**) PDCs were analyzed by flow cytometry as CD123, CD303, CD304, and CD45 positive cells. Representative FACS analysis showing a pDC population in a MM patient; (**b**) PDC frequencies were analyzed in peripheral blood (PB, *n* = 53) and bone marrow (BM, *n* = 10) samples of healthy donors (HD) and compared to newly diagnosed myeloma patients (MM, *n* = 37). Each box shows the median with min and max values. Significant reductions of pDCs were determined as *** *p* < 0.0001 using the non-parametric Mann–Whitney test.

PDC numbers were first determined by expression of CD123, CD303 (BDCA-2), CD304 (Neurophilin-1/BDCA-4), and CD45 (Figure 1a). The activation phenotype was analyzed by the expression of CD45RA and HLA-DR double positive population. Peripheral blood (*n* = 53) and bone marrow (*n* = 10) samples of healthy donors were compared to newly diagnosed myeloma patients (MM, *n* = 37) (Figure 1b). We detected pDCs localized more frequently in BM than PB samples in patients and healthy controls. More importantly, we observed highly significant reductions of pDCs in PB and also in BM of myeloma patients compared to healthy controls (*p* < 0.0001).

Comparative analysis showed a median 0.11% (range 0.02–0.45%) pDCs in MM PB vs. 0.7% (range 0.1–2.5%) in normal PB. Similarly, MM BM showed low counts of pDCs; 0.25% (range 0.05–2.14%) vs. 4.35% (range 2.6–6.8%) in normal BM.

### 3.2. Bone Marrow pDCs in MGUS and Myeloma Patients

**Figure 2:**
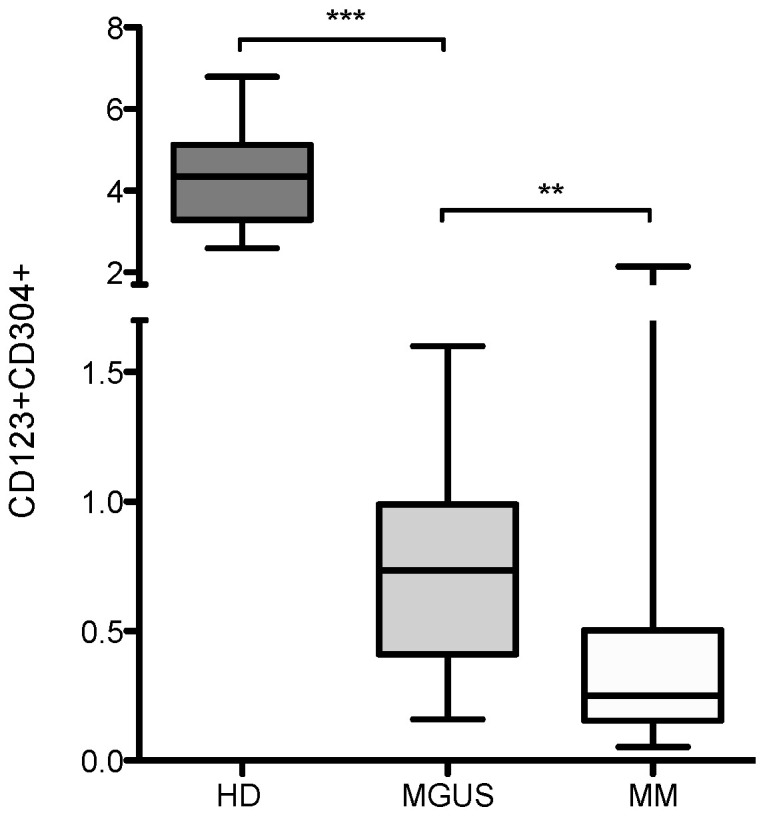
Bone marrow pDCs in MGUS and MM patients. PDC frequencies were analyzed in bone marrow (BM) in healthy donors (HD), MGUS and myeloma patients (MM). Each box shows the median with min and max values. Significant reductions of pDCs were determined as *** *p* < 0.0001 and ** *p* = 0.004 using the non-parametric Mann–Whitney test.

To understand the biology of tumor infiltrating pDCs in the bone marrow microenvironment, we next determined the pDC frequencies in MGUS patients (*n* = 12) and compared to those of myeloma patients (*n* = 37) and healthy donors (*n* = 10) as shown in Figure 2. PDCs in BM MGUS patients were enumerated as 0.7% (range 0.16–1.6%). We observed a significant decrease of pDC levels between HD and MGUS patients (*p* < 0.0001). Furthermore, the prominently reduced pDCs were also found in MGUS vs. MM patients (*p* = 0.004).

### 3.3. Enhanced Proliferation of Myeloma Cells with Co-Cultured pDCs

**Figure 3:**
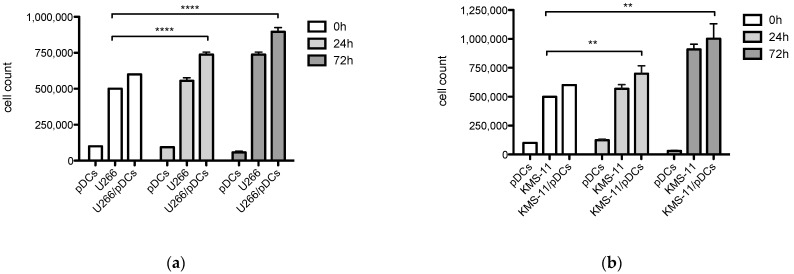

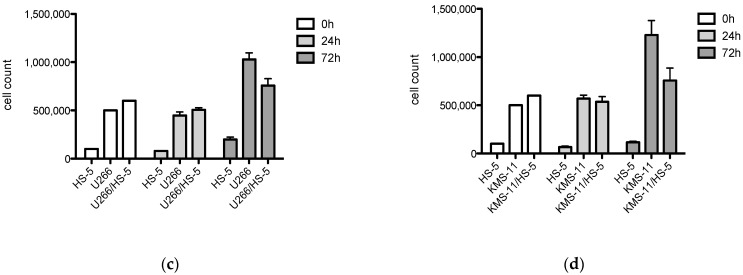
Expansion of myeloma cells in the presence of pDCs: (**a**) Proliferation of MM cell lines including U266 (5 × 10^5^ cells/1 mL); (**b**) KMS-11 (5 × 10^5^ cells/1 mL) alone and in the presence of freshly sorted pDCs (10^5^ cells/1 mL) at a 5:1 ratio for 24 h (pale grey) and 72 h (dark grey) co-cultured in 24-well plates; (**c**) Proliferation of MM cell lines including U266 (5 × 10^5^ cells/1 mL); (**d**) KMS-11 (5 × 10^5^ cells/1 mL) alone and in the presence of the HS-5 cell line (10^5^ cells/1 mL) at a 5:1 ratio for 24 h (pale grey) and 72 h (dark grey) co-cultured in 24-well plates. Data are shown as mean ± SD from three independent experiments. Significant differences in pDC co-cultures were determined using the unpaired *t* test as **** *p* < 0.0001 (U266) and ** *p* = 0.002 and *p* = 0.007 (KMS-11) respectively.

We examined the proliferation rate of MM cells in the presence of freshly isolated pDCs from healthy donors (*n* = 3). The viability and expansion of U266 and KMS-11 myeloma cell lines were determined when co-cultured alone or with pDCs at a 5:1 ratio (MM 5 × 10^5^: pDC 10^5^ cells) for 24 h and 72 h (Figure 3a,b). MM cells were found to prolong survival and proliferate to a significantly greater extent when stimulated in the presence of pDCs in co-cultures with U266 (*p* < 0.0001, at 24 h, and at 72 h) and KMS-11 (*p* = 0.007, 24 h and *p* = 0.002, 72 h). Cultures of pDCs alone showed reduction of viability by 72 h.

To establish whether MM cells were stimulated specifically by pDCs, we tested the viability and expansion of U266 and KMS-11 myeloma cell lines when co-cultured alone or with human stromal HS-5 cells at a 5:1 ratio (MM 5 × 10^5^: HS-5 10^5^ cells) for 24 h and 72 h (Figure 3c,d). Importantly, we found no expansion of MM cell cultures in the presence of HS-5 cells, confirming that pDCs mediated the stimulation of myeloma cell growth.

### 3.4. Production of Intracellular IFNα by pDC

**Figure 4:**
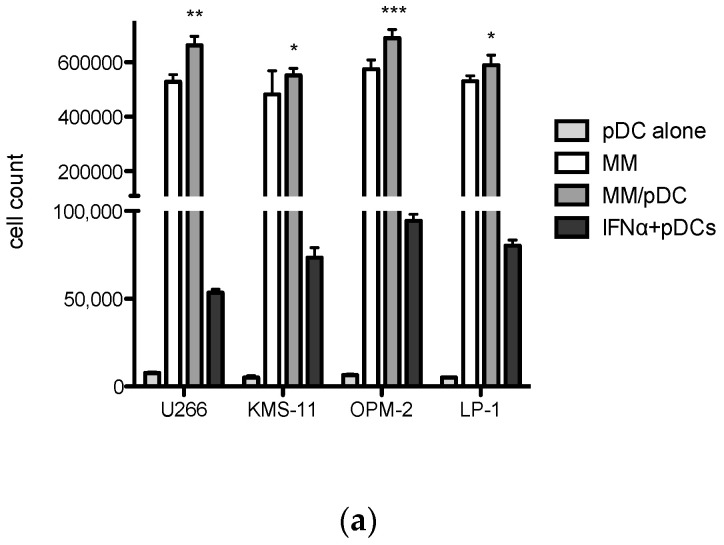

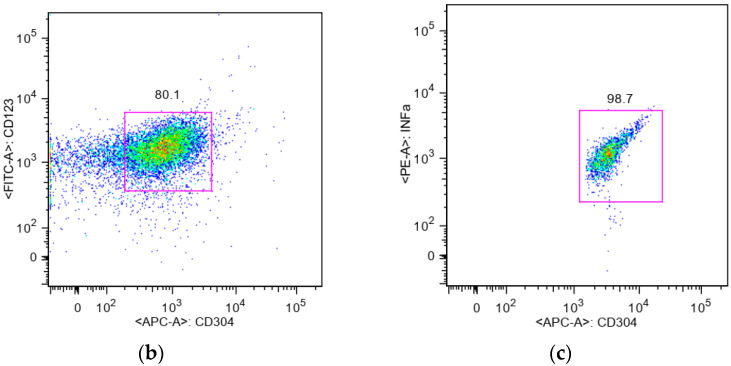
The IFNα secretion by pDCs in the co-cultures with MM cells: (**a**) Proliferation of MM cell lines including U266, KMS-11, OPM-2 and LP-1 (5 × 10^5^ cells/1 mL) alone (white bars) and in the presence of freshly sorted pDCs (10^5^ cells/1 mL) at a 5:1 ratio for 24 h (grey bars) co-cultured in 24-well plates was analyzed. Numbers of IFNα producing pDCs are shown (black bars). Stimulation of pDCs alone with PMA and CI is shown (pale grey bars). Data are shown as mean ± SD from three independent experiments. Significant differences in pDC co-cultures were determined using the unpaired *t* test as * *p* < 0.01 (KMS-11, LP-1), ** *p* = 0.001 (U266) and *** *p* = 0.0005 (OPM-2) respectively. (**b**) Representative FACS plot analysis of pDC populations identified as CD123 and CD304 by surface staining. (**c**) Intracelllular staining showing pDC positive populations secreting IFNα.

Next, we analyzed the IFNα secretion by pDCs in the co-cultures with MM cell lines including U266, KMS-11, OPM2 and LP-1 following 24 h incubation. The myeloma cells (5 × 10^5^) were co-cultured alone or with freshly sorted pDCs from healthy donors (10^5^ cells) and the numbers of IFNα producing pDCs were determined by intracellular staining after 24 h (Figure 4). We found that all four MM cell lines expanded significantly more in the presence of pDCs after 24 h co-culture; U266 (*p* = 0.001), KMS-11 (*p* = 0.01), OPM-2 (*p* = 0.0005) and LP-1 (*p* = 0.01). More importantly, we showed that pDCs produced abundant IFNα as a result of stimulation in the range of 54.3% pDCs+ (U266), 67.9% pDC+ (KMS-11), 95.3% (OPM-2) and 79.7% (LP-1). PDC alone stimulated with PMA and CI showed IFNα secretion below 10%.

## 4. Discussion

In this study, we first described the pDC distributions in newly diagnosed MM and found a profound reduction of pDCs in both peripheral blood and bone marrow samples of MM patients compared to healthy donors. These findings are in an agreement with the early report [25]. However, another report found increased numbers of pDCs in bone marrow from *n* = 32 MM patients using the same FACS analysis, but here the cohort included newly diagnosed patients together with relapsed/refractory disease [26]. It would be of a great interest to split the patient group and identify infiltrated pDCs at diagnosis versus myeloma relapse.

Second, we analyzed bone marrow samples from MGUS patients and also observed a significant decrease in pDCs in BM compared to healthy donors. In addition, highly reduced pDCs were also noted in MM compared to MGUS patients. Importantly, these results suggest a gradual decline of pDCs accumulating in the BM during MGUS to MM progression. The selective decrease in pDC levels may be one of the causes of the reduced capacity of elderly people to initiate antitumor and antiviral immune responses.

Recently, an elegant study suggested that pDCs were proportional to the extent of BM tumor plasma cells in MGUS and MM patients [29]. The authors showed a dual and opposing role of pDCs in MM; in some cases, DCs activated T cells against tumor plasma cells and in others, DCs protected plasma cells from T cell-mediated killing. It has been accepted that in solid tumors including breast [12,13], ovarian [14,15], melanoma [16] and gastric [17] cancers, pDCs were shown to be largely dysfunctional with decreased capacity to produce IFNα and with induction of Tregs that produce IL-10 and TGFβ, which further support tumor progression [10]. Most of the studies concerning the role of pDCs in the tumor microenvironment however lack the functional analysis or the correlation with clinical parameters of the patients.

Third, we found the pathophysiologic functions of pDCs, showing that they promote MM cell proliferation in short-term cultures in contrast to no expansion of MM cell cultures observed in the presence of normal stromal cells. These results confirm that pDCs mediate stimulation of myeloma cell expansion.

Additionally, we showed secretion of IFNα by pDCs upon co-culture with MM tumor cells. Similarly, Chauhan et al. showed that pDCs supported MM tumor cell survival and protected MM cells against bortezomib-induced cytotoxicity [26]. More recent study from this group has identified a novel TLR-9 agonist that inhibited pDC-induced myeloma cell growth and triggered apoptosis, restoring the T cell stimulation [30].

Altogether, our results demonstrate aberrant roles of pDCs in the BM tumor microenvironment in myeloma. Limitations of our study are related to small cohorts of newly diagnosed patients with heterogeneous MM subtypes. It is important to explore the pDC counts in larger cohorts of MGUS, smoldering myeloma and MM patients. In addition, more detailed analysis would be needed to further validate the pDCs function systematically during the MM treatment and patient long-term follow-ups. Future studies should focus on targeting pDC-MM interactions during patient remission and disease relapse at the single cell level. More advances could be made from pDC cell transcriptome profiling that will lead to a better understanding of how myeloma cells educate pDCs in their tumor microenvironment.

Promising future studies focused on pDC biology will use functional assays to elucidate new biomarkers in myeloma including miRNA, angiogenesis markers, extracellular matrix proteins and cfDNA using next-generation sequencing (NGS), mass cytometry CyTOF and CRISPR/Cas9 technologies [31]. Recent identification of E-cadherin expressed on both myeloma cells and pDCs was shown to mediate tumor-promoting properties [32]. Importantly, pDC depletion induced tumor regression in a myeloma mouse model providing pDCs as new targets for improving MM outcomes [32,33]. Aberrant pDC function in MM resulted in decreased T cell proliferation and NK cytolytic activity against tumor cells contributing to immune suppression in MM [32,33]. PDCs are critical for induction of innate and adaptive immune responses and they represent an attractive therapeutic target in anti-tumor immunity [34,35]. Despite the fact that their function within the context of the tumor microenvironment is still not fully known, future perspectives will improve our understanding of pDC responses in cancer patients.

## Figures and Tables

**Table 1:** Summary of patient characteristics.

Parameter		MGUS	MM
Total number, n		12	37
Male		8	20
Female		4	17
Age, years median, range		63, 47–87	68, 45–83
Heavy chain M-Ig (Immunoglobulin)	IgG	7	23
	IgM	1	1
	IgA	1	7
	biclonal	2	-
	LC only	1	5
Light chain M-Ig (Immunoglobulin)	Kappa	6	22
	Lambda	6	14
	biclonal		1
Staging Salmon-Durie	I A		4
	II A		3
	III A		17
	II B		3
	III B		9
			1 Not Available
ISS classification (International Staging System)	Stage 1		11
	Stage 2		10
	Stage 3		14
			2 Not Available
serum M-Ig median, range grams per liter (g/L)		6.2 (0.5–15.4)	28.0 (0–84.9)
Hemoglobin median, range grams per liter (g/L)		139.4 (76.2–161)	102.6 (58–164)
Albumin median, range grams per liter (g/L)		42.6 (36.4–46)	35.8 (17.7–48)
Creatinine median, range micromoles per liter (μmol/L)		114.4 (66–266)	187.9 (53–929)
Beta-2-microglobulin median, range miligrams per liter (mg/L)		2.9 (1.6–6)	8.5 (1.7–40)
% Plasma cells Bone marrow infiltration median, range		2.5 (0.4–7.6)	28.6 (4.2–76)

## Data Availability

Data are available upon request. All data relevant to the study are included in the article.
